# TGF-β – an excellent servant but a bad master

**DOI:** 10.1186/1479-5876-10-183

**Published:** 2012-09-03

**Authors:** Lenka Kubiczkova, Lenka Sedlarikova, Roman Hajek, Sabina Sevcikova

**Affiliations:** 1 Babak Myeloma Group, Department of Pathological Physiology, Faculty of Medicine, Masaryk University, Brno, 625 00, Czech Republic

**Keywords:** TGF-β, SMAD proteins, Oncogene, Suppressor, Solid tumors, Leukemia, Multiple myeloma

## Abstract

The transforming growth factor (TGF-β) family of growth factors controls an immense number of cellular responses and figures prominently in development and homeostasis of most human tissues. Work over the past decades has revealed significant insight into the TGF-β signal transduction network, such as activation of serine/threonine receptors through ligand binding, activation of SMAD proteins through phosphorylation, regulation of target genes expression in association with DNA-binding partners and regulation of SMAD activity and degradation. Disruption of the TGF-β pathway has been implicated in many human diseases, including solid and hematopoietic tumors. As a potent inhibitor of cell proliferation, TGF-β acts as a tumor suppressor; however in tumor cells, TGF-β looses anti-proliferative response and become an oncogenic factor. This article reviews current understanding of TGF-β signaling and different mechanisms that lead to its impairment in various solid tumors and hematological malignancies.

## Introduction

Although our understanding of molecular mechanisms that underlie cancer development and progression has increased, cancer remains a significant health concern in many developed countries. There is a strong requirement for new diagnostic and treatment options as well as elucidation of how cells acquire the six essential phenotypes, or hallmarks, necessary to become fully malignant [1]. Pharmacological targeting of cancer hallmarks may offer new possibilities of effectively treating development and/or metastases of human tumors (reviewed in [2]). Transforming Growth Factor-β (TGF-β) is a key player in cell proliferation, differentiation and apoptosis. The importance of this regulation is apparent from the role of TGF-β in development and consequences of aberrant TGF-β signaling in cancer [3]. Nevertheless, it is still not elucidated how malignant cells overcome the cytostatic functions of TGF-β or how TGF-β stimulates the acquisition of cancer hallmarks of developing and progressing human cancers. In this paper, we review different molecular and cellular mechanisms that lead to impairment of TGF-β signaling in various solid tumors and hematological malignancies.

## History of TGF-β discovery

In the early 1980s, it had become apparent that cell growth is controlled by many polypeptides and hormones. A new hypothesis of ‘autocrine secretion’ was postulated, which suggested that polypeptide growth factors are able to cause malignant transformation of cells [4]. A new polypeptide called SGF (Sarcoma Growth Factor) was discovered in cultures of transformed rat kidney fibroblasts [5]; soon it became apparent that this factor is a mixture of at least two substances with different functions. They were called Transforming Growth Factor-α (TGF-α) and Transforming Growth Factor-β (TGF-β) [6]. TGF-β was further described by Roberts and Sporn as a secreted polypeptide capable of inducing fibroblast growth and collagen production [7]. Soon after its discovery, TGF-β was found to inhibit cell proliferation as well; thus, a dual role of this cytokine was recognized [8,9].

### TGF-β family and isoforms

The TGF-β superfamily is composed of a large group of proteins, including the activin/inhibin family, bone morphogenetic proteins (BMPs), growth differentiation factors (GDFs), the TGF-β subfamily, and the glial cell line-derived neurotrophic factor (GDNF) family. This review will focus solely on the TGF-β family.

The TGF-β proteins have been discovered in a variety of species, including invertebrates as well as vertebrates. TGF-β superfamily is fundamental in regulation of various biological processes, such as growth, development, tissue homeostasis and regulation of the immune system [10,11].

Beta-type subfamily growth factors are homodimeric or heterodimeric polypeptides with multiple regulatory properties depending on cell type, growth conditions and presence of other polypeptide growth factors. Since their expression is also controlled by distinct promoters, their secretion is temporal and tissue specific [12].

There are three known isoforms of TGF-β (TGF-β1, TGF-β2 and TGF-β3) expressed in mammalian tissues; they contain highly conserved regions but diverge in several amino acid regions. All of them function through the same receptor signaling pathways [13,14].

TGF-β1, the most abundant and ubiquitously expressed isoform, was cloned from human term placenta mRNA [15]. In mouse development, Tgf-β1 mRNA and/or protein have been localized in cartilage, endochondral and membrane bone and skin, suggesting a role in the growth and differentiation of these tissues [16].

TGF-β2 was first described in human glioblastoma cells. It was found that TGF-β2 is capable of suppressing interleukin-2-dependent growth of T lymphocytes. Thereby, it was named glioblastoma-derived T cell suppressor factor (G-TsF). Physiologically, TGF-β2 is expressed by neurons and astroglial cells in embryonic nervous system [17]. It is also important in tumor growth enhancing cell proliferation in an autocrine way and/or reducing immune-surveillance of tumor development [18]. Their mature forms, which consist of the C-terminal 112 amino acids, TGF-β1 and TGF-β2 share 71% sequence similarity [19].

The third isoform, TGF-β3, was isolated from a cDNA library of human rhabdomyosarcoma cell line; it shares 80% of amino acid sequence with TGF-β1 and TGF-β2. Studies on mice demonstrated essential function of Tgf-β3 in normal palate and lung morphogenesis and implicate this cytokine in epithelial-mesenchymal interaction [20,21]. Its mRNA is present in lung adenocarcinoma and kidney carcinoma cell lines; interestingly, umbilical cord expresses very high level of TGF-β3 [19].

### TGF-β synthesis and activation

**Figure 1:**
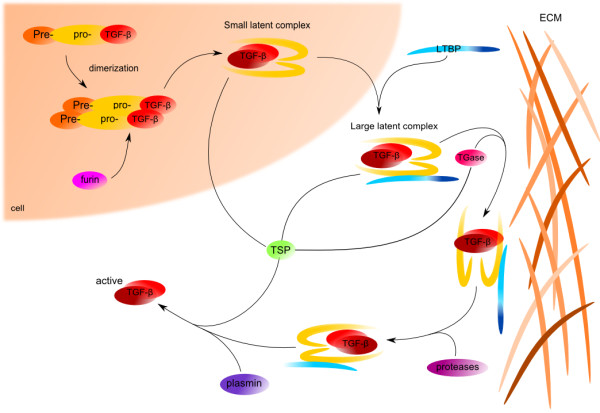
**TGF-β synthesis and activation.** TGF-βs are synthesized as inactive precursors that contain pre-region (Signal peptide) and pro-region (N terminal peptide - LAP). Processing of inactive form starts with proteolytic cleavage that removes signal peptide from pre-pro-TGF-βs form. After dimerization, TGF-βs are cleaved by proteases (eg. Furin) into C-terminal mature peptides and N-terminal LAP (Latency Associated Peptide). TGF-βs with LAP form small latent complexes (SLP) that are transported to extracellular matrix where can further covalently bind to latent TGF-β binding protein (LTBP) to form a large latent complexes (LLC). LTBP is able to connect inactive TGF-β forms to ECM proteins. This interaction is further supported by covalent transglutaminase-induced (TGase) crosslinks. Activation of TGF-β starts with release of LCC from ECM by proteases. Then, the mature protein is cleaved from LTBP, which is provided *in vitro* by acidic condition, pH or plasmin or *in vivo* by thrombospondin (TSP). Once the active TGF-β family member is released from the ECM, it is capable of signaling

Mature dimeric form of TGF-β, composed of two monomers stabilized by hydrophobic interactions and disulphide bridge, initiates intracellular signaling [22]. The three TGF-βs are synthesized as pro-proteins (pro-TGF-βs) with large amino-terminal pro-domains (called latency associated proteins – LAPs), which are required for proper folding and dimerization of carboxy-terminal growth-factor domain (mature peptide) [23]. This complex is called ‘small latent complex’ (SLC). After folding and dimerization, TGF-β dimer is cleaved from its pro-peptides in trans-Golgi apparatus by furin type enzymes; however, it remains associated with its pro-peptide through noncovalent interactions, creating ‘large latent complex‘ (LLC). Most cultured cell types release latent TGF-β into extracellular matrix as LLC which in addition includes a 120–240 kDa glycoprotein called latent TGF-β binding protein (LTBP) [24]. LTBP is composed primarily of two kinds of cysteine-rich domains: EGF-like repeats (most of which are calcium-binding) and eight-cysteine domains [25]. LTBP participates in the regulation of latent TGF-β bioavailability by addressing it to the extracellular matrix (ECM) [26]. Non-active TGF-β stays in ECM; its further activation is a critical step in the regulation of its activity (Figure 1). 

A number of papers have reported TGF-β activation by retinoic acid and fibroblast growth factor-2 (FGF-2) in endothelial cells [27,28], or by endotoxin and bleomycin in macrophages [29]. Further, a variety of molecules is involved in TGF-β activation. Proteases including plasmin, matrix metaloproteases MMP-2 and MMP-9, are TGF-β activators *in vitro*[30,31]. Other molecules involved in the mechanism of activation are thrombospondin-1 [32], integrins, such as αVβ6 or αVβ8 [33,34], or reactive oxygen species (ROS).

Moreover, latent TGF-β present in conditional medium is activated by acid treatment (pH 4.5) *in vitro*[35]. *In vivo*, a similar pH is generated by osteoclasts during bone resorption. Since the bone matrix deposited by osteoblasts is rich in latent TGF-β, the acidic environment created by osteoclasts *in vitro* might result in latent TGF-β activation [36].

## TGF-β receptors

In most cells, three types of cell surface proteins mediate TGF-β signaling: TGF-β receptor I (TβRI), II (TβRII) and III (TβRIII) [13,37]. Out of these three receptors, TβRIII, also called betaglycan, is the largest (250–350 kDa) and most abundant binding molecule. This cell-surface chondroitin sulfate / heparan sulfate proteoglycan is expressed on both fetal and adult tissues and most cell types [38]. Endoglin (CD105) was shown to act as type III receptor for TGF-β as well [39]. Endoglin is a membrane, an RGD-containing glycoprotein, which is expressed in a limited set of cell types, primarily vascular endothelial cells, several hematopoietic cell types, bone marrow stromal cells and chondrocytes. Its expression strongly increases in active vascular endothelial cells upon tumor angiogenesis [40-42]. Moreover, in normal brain, it was found to be expressed in the adventitia of arteries and arterioles, and it is expressed on several types of tumor cells, such as invasive breast cancers and cell lines or renal cell carcinoma [43-45]. Although betaglycan and endoglin are co-receptors not directly involved in intracellular TGF-β signaling due to lack of kinase domain, they can control access of TGF-β to TGF-β receptors and consequently modulate intracellular TGF-β activity [46,47]. Betaglycan binds all three isoforms of TGF-β, with higher affinity for TGF*-*β2; however, endoglin binds TGF-β1 and -β3 with constant affinity and has only weak affinity for TGF-β2 [39,48].

TβRI and TβRII mediate signal transduction. Both receptors are transmembrane serine/theronine kinases, which associate in a homo- or heteromeric complex and act as tetramers. They are organized sequentially into an N-terminal extracellular ligand-binding domain, a transmembrane region, and a C-terminal serine/threonine kinase domain. The type II receptors range from 85 to 110 kDa, while the type I receptors are smaller and their size ranges from 65 to 70 kDa [49]. Moreover, TβRI contains a characteristic, highly conserved 30 amino acids long GS domain in the cytoplasmic part, which needs to be phosphorylated to fully activate TβRI [36]. TβRII contains 10 bp polyadenine repeat in the coding region of the extracellular domain. This region is frequently a target of changes leading to frameshift missense mutations or early protein terminations that result in truncated or inactive products [50].

### TGF-β receptors activation

Bioactive forms of TGF-βs are dimers held together by hydrophobic interactions and, in most cases, by an intersubunit disulfide bond as well. The dimeric structure of these ligands suggests that they function by bringing together pairs of type I and II receptors, forming heterotetrameric receptor complexes [51]. Binding of TGF-β to extracellular domains of both receptors also induces proper conformation of the intracellular kinase domains. These receptors are subject to reversible post-translational modifications (phosphorylation, ubiquitylation and sumoylation) that regulate stability and availability of receptors as well as SMAD and non-SMAD pathway activation.

Receptor phosphorylation activates TGF-β signaling pathway – the ligand binds to TβRII first, followed by subsequent phosphorylation of a Gly-Ser regulatory region (GS-domain) within TβRI. This leads to incorporation of TβRI and formation of a large ligand-receptor complex that consists of dimeric TGF-β ligand and two pairs of TβRI and TβRII [52]. The TGF-β receptor complex is extremely stable upon solubilization [53]. TGF-β1 and TGF-β3 bind to TβRII without participation of type I receptor, whereas TGF-β2 interacts only with combination of both receptors (reviewed in [54]). Although ligand binding may induce autophosphorylation of TβRII cytoplasmic domain, signaling in the absence of TβRI has not been reported [49]. TβRIII betaglycan promotes binding of TGF-β2 to TβRII, since the affinity of TGF-β2 to TβRII is low in the absence of betaglycan [46]. Endoglin binds TGF-β1, TGF-β3 but not TGF-β2 in the presence of the TβRI and TβRII. In some cell types, endoglin was found to inhibit TGF-β signaling – for example in chondrocytes, it enhances TGF-β1-induced SMAD1/5 phosphorylation but inhibits TGF-β1-induced SMAD2 phosphorylation [55].

Ubiquitylation and ubiquitin-mediated degradation define stability and turnover of receptors. Ubiquitylation occurs through sequential actions of E1, E2 and E3 ubiquitin ligases that provide specificity in the ubiquitylation process [56]. The E3 ubiquitin ligases such as Smurf1 and Smurf2 (SMAD ubiquitylation-related factor 1 and 2) regulate the stability of TβRI and heteromeric TGF-β receptor complex [57,58].

Sumoylation, similarly to ubiquitylation, requires E1, E2 and E3 ligases which results in SUMO polypeptide attachment. Although sumoylation has not been observed for any other transmembrane receptor kinases, it was shown to modify TβRI function by facilitating the recruitment and phosphorylation of SMAD3 [59].

TGF-β receptors are also constitutively internalized via clathrin-dependent or lipid-raft-dependent endocytic pathways (reviewed in [60]).

## TGF-β signaling

### SMAD proteins

The SMAD proteins are the only known latent cytoplasmic transcription factors that become directly activated by serine phosphorylation at their cognate receptors. SMADs can be classified into 3 groups based on their function: the receptor-regulated SMADs (R-SMADs), SMAD1, SMAD2, SMAD3, SMAD5 and SMAD8; the common SMAD (Co-SMAD), SMAD4, and the inhibitory SMADs (I-SMADs), SMAD6 and SMAD7 (reviewed in [61]).

R-SMADs and Co-SMAD consist of a conserved MH1 domain (Mad-homology-1) and C-terminal MH2 domain (Mad-homology-2), which are connected by a ’linker’ segment. The C-terminal domain promotes transcriptional activity, when fused to a heterologous DNA binding domain [62]. On the contrary, I-SMADs contain only the highly conserved MH2 domain. The MH1 domain is responsible for binding to DNA; however, the MH2 domain contains hydrophobic patches also called hydrophobic corridors that allow binding to nucleoporins, DNA-binding cofactors and various cytoplasmic proteins, as well as interaction with receptors. Both domains can interact with sequence-specific transcription factors. SMAD3 and SMAD4 bind with their MH1 domain to SMAD-binding elements (SBE) on DNA, whereas the common splice form of SMAD2 does not bind to DNA (reviewed in [63]).

I-SMADs function as intracellular antagonists of R-SMADs. Through stable interactions with activated serine/threonine receptors, they inhibit TGF-β family signaling by preventing the activation of R- and Co-SMADs. I-SMADs regulate activation of R-SMADs via binding with their MH2 domain to TβRI, thereby competing with R-SMADs and preventing R-SMADs phosphorylation [64]. SMAD6 is also able to compete with SMAD4 for heteromeric complex formation with activated SMAD1 [65]. Whereas SMAD6 appears to preferentially inhibit BMP signaling, SMAD7 acts as a general inhibitor of TGF-β family signaling. Another possible mechanism of inhibition signaling transduction by I-SMADs is facilitated by HECT type of E3 ubiquitin ligase Smurf1 and Smurf2 [57,58].

### Canonical signaling

**Figure 2:**
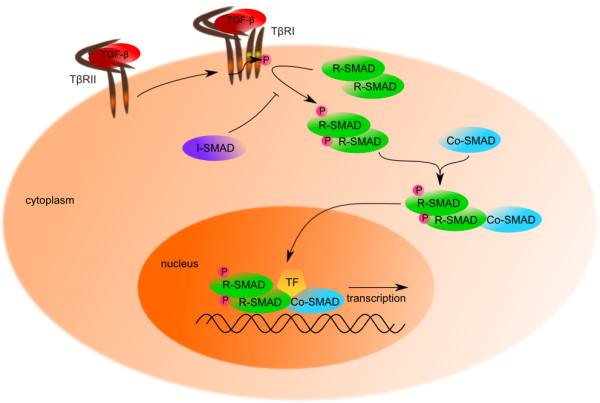
**TGF-β canonical signaling pathway.** After ligand binding, TGF-β receptors dimerize and phosphorylate intracellular SMAD proteins. Complex of SMAD2 and/or SMAD3 becomes phosphorylated by TβRI and forms a complex with SMAD4 which is subsequently transported into the nucleus where it binds with specific transcription factors (TF) and induces a transcription of TGF-β dependent genes

The SMAD pathway is the *canonical signaling* pathway that is activated directly by the TGF-β cytokines (Figure 2). TβRI recognizes and phosphorylates signaling effectors – the SMAD proteins. This phosphorylation is a pivotal event in the initiation of TGF-β signal, followed by other steps of signal transduction, subjected to both positive and negative regulation.

R-SMAD binding to the type I receptor is mediated by a zinc double finger FYVE domain containing protein SARA (The SMAD Anchor for Receptor Activation). SARA recruits non-activated SMADs to the activated TGF-β receptor complex [66]. However, TMEPAI (TransMembranE Prostate Androgen-Induced gene/protein), a direct target gene of TGF-β signaling, perturbs recruitment of SMAD2/3 to TβRI and thereby participates in a negative feedback loop to control the duration and intensity of SMADs activation [67]. Receptor-mediated phosphorylation of SMAD2 decreases the affinity of SMAD2 to SARA, leading to dissociation from SARA [68]. Afterwards, phosphorylated complex of SMAD2/3 forms a higher-order complex with SMAD4 and moves to the nucleus. At this point, Smurf1 interacts with R-SMADs in order to trigger their ubiquitylation and degradation and hence their inactivation [69]. Further, it was found that Smurf1 and Smurf2 facilitate the inhibitory effect of I-SMADs. Smurf2 binding in the nucleus to SMAD7 induces export and recruitment to the activated TβRs, where it causes degradation of receptors and SMAD7 via proteasomal and lysosomal pathways [57]. Smurf1 (specific for BMP-SMADs) also interacts with SMAD7 and induces SMAD7 ubiquitylation and translocation into the cytoplasm [58].

For proper translocation to the nucleus, the SMADs contain a nuclear localization-like sequence (NLS-like; Lys-Lys-Leu-Lys) that is recognized by importins [70]. Interestingly, the nuclear translocation of SMADs was also described *in vitro* to occur independently of added importin-like factors, because SMAD proteins can directly interact with nucleoporins, such as CAN/Nup214 [71,72]. Complex of SMAD2/3 and SMAD4 is retained in the nucleus by interactions with additional protein binding partners and DNA. Dephosphorylation and dissociation of SMAD transcriptional complexes are thought to end this retention, allowing export of R-SMADs out of the nucleus [73].

Different protein binding partners provide another venue for regulatory inputs controlling the activity of SMADs. Each SMAD-partner combination targets a particular subset of genes and recruits either transcriptional co-activators or co-repressors. Members of many DNA-binding protein families participate as SMADs cofactors, such as FOX, HOX, RUNX, E2F, AP1, CREB/ATF, Zinc-finger and other families. The SMAD cofactors differ in various cell types, thereby determining the cell-type dependent responses [63]. By association with DNA-binding cofactors, SMADs reach target gene specificity and target specificity. Stimulation of various cells by TGF-β leads to rapid activation or repression of a few hundred genes; possibly, the pool of activated SMAD proteins is shared among different partner cofactors [74,75].

On chromatin level, SMADs can recruit histone acetyltransferases. Several studies revealed that TGF-β proteins influence transcription of different genes through interaction of the MH1 domain of SMADs with sequence-specific transcription factors and co-activators CBP and p300. CBP and p300 interact with SMAD1, SMAD2, SMAD3 and SMAD4 *in vitro* and *in vivo*, and the interaction between the SMADs and CBP/p300 is stimulated in response to TGF-β [76-79]. Moreover, histone deacetylases and chromatin remodeling complexes are also involved in SMAD regulation. In this way, SMADs functionally interact with a variety of transcription factors and regulate diverse signaling pathways as well (reviewed in [80]).

SMADs act as sequence specific transcription factors; however, they can regulate cell fate by alternative mechanisms. Recent data indicate that R-SMADs associate with the p68/Drosha/DGCR8 miRNA processing complex to regulate miRNA processing in a ligand-dependent and RNA-sequence specific manner. So far, more than 20 TGF-β/BMP-regulated miRNAs (T/B-miRs) have been described [81,82].

### Non-SMAD signaling

**Figure 3:**
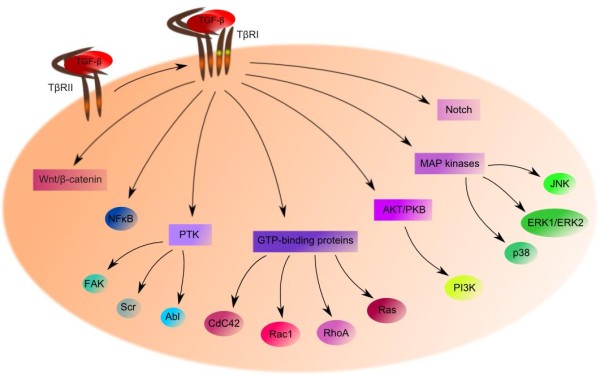
**TGF-β non-canonical signaling pathway.** After ligand binding, several different branching signaling pathways can be activated in malignant cells, such as Notch signaling, MAP kinases, AKT/PKB pathway, GTP-binding proteins pathway, PTK pathway, NF-κB and Wnt/β-catenin pathway

Diversity of TGF-β signaling in cells is determined not only by various ligands, receptors, SMAD mediators or SMAD-interacting partners, but also by the ability of TGF-β to activate other signaling pathways (Figure 3). TGF-β can indirectly participate in apoptosis, epithelial to mesenchymal transition, migration, proliferation, differentiation and matrix formation (reviewed in [83]). It activates various branches of mitogen-activated protein kinases (MAPK) pathway, such as ERK1/ERK2, Jun-N terminal kinase (JNK) and p38 and PI3K kinases [84]. In response to TGF-β, both SMAD-dependent and SMAD-independent JNK activations are observed [85]. SMAD-independent activation of p38 was observed in mouse mammary epithelial NMuMG cells with mutant TβRI [86]. 

Other pathways influenced by TGF-β are the growth and survival promoting pathway AKT/PKB, the small GTP-binding proteins RAS, RHOA, RAC1 as well as CDC42 and mTOR [87-89]. TGF-β participates in mediating activation of protein tyrosine kinases FAK, SRC and ABL, particularly in mesenchymal or dedifferentiated epithelial cells [90-92]. TGF-β also influences NF-κB signaling and Wnt/β-catenin pathway [93].

## Role of TGF-β in tumors

In tumors, TGF-β can be either a proto-oncogene or a tumor suppressor, depending on cell context and tumor stage [94]. Cancer cells often evade growth inhibition effects of TGF-β, while leaving intact TGF-β-mediated cellular responses that promote tumor progression.

Importantly, the use of mouse models has enabled the elucidation of the dual role of TGF-β in cancer (reviewed in [95]). As homozygous deletions of *Tgf-β1, Tgf-β2, Tgf-β3, TβRI* and *TβRII* are lethal in mice, manipulation of TGF-β pathway was achieved mainly through transgene expression or conditional null mutations *in vivo*[96]. The dual role of TGF-β was shown on a set of experiments with mice skin cancer. The first study demonstrated that TGF-β1 expression targeted to keratinocytes inhibits benign tumor outgrowth; however, later it enhances malignant progression rate and phenotype of the benign papillomas [97]. Study on transgenic mice overexpressing a dominant negative TβRII in the basal cell compartment and in follicular cells of the skin complemented previous results. In non-irritated epidermis of transgenic mice, proliferation and differentiation were normal; however, during tumor promotion, transgenic mice showed an elevated level of proliferation in the epidermis [98]. Furthermore, using mice with inducible expression of TGF-β1 in epidermis confirmed the dual role of TGF-β [99,100].

### TGF-β as a tumor suppressor

The most critical effect of TGF-β on target cells is suppression of proliferation. Its growth inhibitory function is based on the ability to suppress expression and function of c-Myc and cyclin-dependent kinases (CDKs) and to enhance expression of the CDK inhibitors p15^INK4B^[101][102] and p27^KIP1^[103].

**Figure 4:**
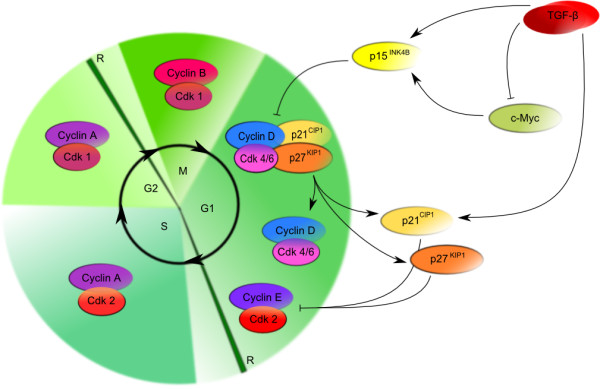
**Role of TGF-β in regulation of cell cycle.** Physiologically, TGF-β is a potent inhibitor of cell cycle; it induces expression of p15^INK4B^ and represses expression of c-Myc. p15^INK4B^ is able to prevent cyclin D-CDK4/6 complex formation; moreover, it displaces p21^CIP1^ and p27^KIP1^ from cyclin D-CDK4/6 complexes. These CIP/KIP inhibitors are subsequently able to inactivate other complexes of G1 and S phase and thereby inhibit cell cycle. Moreover, low levels of c-Myc allows for TGF-β induced p15^INK4B^ and p21^CIP1^ transcription

Cellular responses to TGF-β depend on cell type and physiological conditions. TGF-β stimulates various mesenchymal cell types, including fibroblasts; however, it is a potent inhibitor of epithelial, endothelial, neural cells and hematopoietic cells, including immune cells [10]. Central function of TGF-β is inhibition of cell cycle progression by regulating transcription of cell cycle regulators (Figure 4). Anti-proliferative responses can be induced at any time during cell cycle division; yet, they are effective only in G1 phase. Once a cell is committed to enter replication, it will continue to double its DNA, divide and then arrest when entering the following G1 phase. At this point, TGF-β mediates cell cycle arrest by suppressing expression and function of c-Myc, members of the Id family inhibitors and CDKs and enhancing expression of CDK inhibitors, such as p15^INK4B^, p21^CIP1^ and p27^KIP1^[104,105]. 

TGF-β induces the expression of the CDK inhibitor p15^INK4B^ in a variety of cell types. p15^INK4B^ is a member of the INK4 family of CDK inhibitors, which binds to CDK4 and CDK6 subunits, inactivates their catalytic activity and prevents cyclin D-CDK4/6 complex formation [101,106]. Furthermore, TGF-β can induce expression of p21^CIP1^ in several cell types [107,108]. Other CDK inhibitory responses, observed in several cell types after exposure to TGF-β, are inhibition of CDK4 expression and down-regulation of CDC25A expression [109].

Low levels of c-Myc allow for TGF-β induced transcription of p15^INK4B^ and p21^CIP1^ genes. Decreased expression of c-Myc in keratinocytes is mediated by SMAD3 in association with transcription factors E2F4 and E2F5, p107 co-repressor and SMAD4 [110]. On the other hand, down-regulation of Id proteins in epithelial cells is due to activated SMAD3 that induces activating transcription factor (ATF) expression and then together with ATF directly represses the Id promoter [104].

### TGF-β as a tumor promoter

TGF-β acts as tumor suppressor in normal epithelium; it inhibits cell proliferation and induces apoptosis. Yet, during tumor progression, sensitivity to these effects of TGF-β is frequently lost and, in later stages, TGF-β signaling has pro-oncogenic function. Several activities have been described to TGF-β that would favor tumor progression [111].

### Mutations in signaling components

Malignant cells become resistant to suppressive effects of TGF-β either through mutation and/or functional inactivation of TGF-β receptors or by downstream alterations in the SMAD-signaling pathway. During late stages of tumor progression, TGF-β acts as tumor promoter and is often over-expressed in many cancers. Elevated plasma level of TGF-β1 was observed in hepatocellular carcinoma, colon, HCC, prostate, lung and breast cancers and correlates with poor prognosis [112].

Mutations in downstream TGF-β signaling components cause variable attenuations or complete loss of expression; these mutations, which have been detected in many common tumors, affect TGF-β signal transmission that potentially results in human cancer development and progression. In particular, TβRI, TβRII, SMAD2 and SMAD4 are frequently lost, mutated or attenuated (gene/LOH/expression). Inactivation of TβRII leads to increased tumor spreading and metastasis in a variety of carcinomas, including colon [113], breast [114], pancreatic [115], intestinal [116] or head and neck squamous cell carcinoma (HNSCC) [117]. Also, deregulated expression or aberrant function of Smurf1 and 2 was described. Several human carcinoma cell lines such as colon HT-29, breast MDA-MB-231, gastric MKN-1 and ovarian OVCAR-5 display high levels of one or more E3 ligases, including Smurf2 [118,119]. Moreover, in esophageal squamous carcinoma, high expression levels of Smurf2 associated with low levels of SMAD2 phosphorylation were detected [120]. Furthermore, TGF-β pathway is modulated by epigenetic mechanisms, such as transcriptional repression of *TβRII*, DNA methylation of *TβRI* and *TβRII* and histone modifications [121-123].

### TGF-β in tumor microenvironment and metastases

Tumor metastases accounts for the majority of cancer associated deaths. Recent evidence strongly suggests that tumor microenvironment is essential in this process. It consists of tumor cells and a variety of immune cells, which infiltrate into tumors. This dynamic microenvironment is not only important for cross-talk with tumor cells or escape of tumor from host immune surveillance, but it also induces formation of new blood vessels and invades the vasculature. Areas of hypoxic tissue are thought to drive genomic instability and alter DNA damage repair [124]. Recent studies suggest that TGF-β is one of the critical regulators of inflammation; it is thought that tumor metastasis is a coordinated process between tumor cells and host cells through inflammation [125]. However, it seems that different mechanisms are implemented in different tumor type.

TGF-β as a proto-oncogene is important in stromal-epithelial cross-talk, as was shown for the first time in mouse experiments, where deletion of the TβRII in stromal fibroblasts resulted in transformation of adjacent epithelia of prostate and forestomach. Moreover, in this model, hepatocyte growth factor (HGF) was up-regulated and complementary activation of the HGF receptor MET was detected in tissues where TβRII had been ablated, which implicates this paracrine signaling network as a potential mechanism for regulation of carcinoma development [126]. Further experiment performed on these mice revealed that mice fibroblasts have up-regulated expression of growth factors and increased proliferation of mammary cancer cells [127]. Together, it indicates that TGF-β responses mediated by stromal fibroblasts can regulate carcinoma initiation and progression of adjacent epithelium *in vivo* and *in vitro.*

Interestingly, it was found that TGF-β in breast cancer favors metastasis to lungs. TGF-β stimulation of mammary carcinoma cells in tumor microenvironment, before they enter circulation, primes these cells for seeding of lungs through a transient induction of angiopoetin-like4 (Angptl4) via canonical signaling pathway [128]. TGF-β is involved in regulation of chemokines and chemokine receptors which take part in inflammatory cells recruitment. The loss of TβRII in breast cancer cells can enhance recruitment of F4/80^+^ cells to tumor microenvironment and increase the expression of pro-inflammatory genes, including *CXCL1, CXCL5* and *PTGS2* (cyclooxygenase-2). Further, *in vitro* treatment of carcinoma cells with TGF-β suppressed the expression of *CXCL1, CXCL5* and *PTGS2*[129].

Different mechanism was observed in gastric carcinoma, where SMAD-dependent TGF-β pathway, in collaboration with PKC-δ expression and phosphorylation and integrin expression and activation, regulates cell invasion and cell spreading [130].

Beside the effects already mentioned, TGF-β is broadly implemented in induction of epithelial-to-mesenchymal transition [131]. The NBT-II cell line, derived from a chemically induced rat bladder carcinoma, forms epithelial colonies that can be converted into migratory mesenchymal cells within a few hours by adding Tgf-β and other factors, such as Fgf1, Fgf7, Fgf10, Egf, Igf1, Igf2 or Hgf [132].

### TGF-β as a regulator of immune cells

The tumor microenvironment is filled with various inflammatory cells, including myeloid cell subpopulations, T cells and B cells. TGF-β is one of the most potent endogenous negative regulators of hematopoiesis. It modulates proliferation, differentiation and function of all types of lymphocytes, macrophages and dendritic cells, thus regulating the innate, non-antigen-specific as well as antigen-specific immunity [133].

TGF-β is involved in normal B cells maturation and differentiation, such as regulation of expression of cell-surface molecules, inhibition of IgM, IgD, CD23 and the transferrin receptor and induction of MHC class II expression on pre-B cells and mature B cells [134].

In T cells, TGF-β regulates maturation; for example, it is released by regulatory T cells and inhibits the Ag-specific proliferation of naive CD4+ cells from T cell receptor (TCR) [135]. TGF-β1 also inhibits aberrant T cell expansion by maintaining intracellular calcium concentration levels low enough to prevent mitogenic response by Ca^2+^-independent stimulatory pathways [136].

In myeloid cells, such as macrophages and monocytes, TGF-β1 is mostly suppressive, it inhibits cell proliferation and down-regulates production of reactive oxygen and nitrogen intermediates; however, it is able to enhance some other activities of myeloid cells. TGF-β1 can be recognized by monocytes and macrophages as a chemotactic factor; it induces direct monocytes migration *in vitro*[137].

TGF-β pro-metastatic and pro-inflammatory effects are regulated via nuclear factor kappa B (NF-κB), the master regulator of inflammation and a regulator of genes that controls cell proliferation and cell survival. TGF-β1 is a negative regulator of NF-κB activation, as was shown in the gut; it directly stimulates IκB-α promoter transcriptional activity *in vitro*. However, SMAD7 maintains high NF-κB activity by blocking TGF-β1 signaling [138].

### Targeting the TGF-β signaling pathway

As the signaling pathway deregulations are responsible for cancer initiation and progression, interrupting the tumor promoter properties of TGF-β signaling would be an attractive therapeutic strategy, without altering physiologic tumor suppressor functions exhibited in early stages of tumorigenesis. Strategies such as using monoclonal TGF-β-neutralizing antibodies, large molecule ligand traps, reducing translational efficiency of TGF-β ligands using antisense technology and antagonizing TGF-β receptor I/II kinase function by small molecule inhibitors are the most prominent methods being explored today [139,140]. Furthermore, studies have shown that combined treatment with tumor cell vaccines and antisense TGF-β therapy reduced tumor size and increased survival benefit [141,142]. Preclinical studies also show that TGF-β inhibition can augment therapeutic efficacy of cytotoxic agents [143]. However, as there are still potential limitations and risks of TGF-β targeted therapy, caution must be given as to when, how and how much therapy would be beneficial or how much toxicity will be induced by chronically administered therapy. However, daily administration of a high dose of neutralizing TGF-β antibody in adult mice for 12 weeks and a lifetime exposure to soluble TβRII (sTβRII) in transgenic mice did not significantly affect their health. This suggests that anti-TGF-β treatments are likely to be safe [144].

## TGF-β in solid tumors

### Brain tumors

TGF-β has a suppressive role in physiological development of the central nervous system (CNS): all TGF-β isoforms and receptors necessary for TGF-β signal transduction are detected in developing as well as adult CNS [145].

The most aggressive type of primary brain tumors, glioblastoma multiforme (GBM), is characterized by poorly differentiated and highly proliferating cells that originate from glial cells [146,147]. Here, the release from cytostatic TGF-β effect is explained by a broad range of inactivating mutations in the TGF-β signaling pathway. Several studies describe mutations in TβRI and TβRII in adenomas and gliomas [148,149] as well as correlation between higher expression of TβRI and TβRII with more aggressive glioma cell lines and tumors [150,151]. Moreover, high levels of TGF-β indicate that TGF-β is able to induce its own expression and thereby create a malignant autocrine loop and control glioma-cell proliferation [152]. Alterations of SMAD protein levels and activation were reported in brain tumor cell lines and patient samples. In glioma cell lines, SMAD3 level and SMAD2 nuclear translocation was lower in 9 out of 10 cell lines [153]. Kjellman *et al.* reported that SMAD2, SMAD3 and SMAD4 mRNA levels were reduced in GBM samples in comparison to normal brain samples, astrocytomas and anaplastic astrocytomas [150]. Nevertheless, these data are controversial to a study in which higher phospho-SMAD2 (p-SMAD2) level correlated with higher grade of glioma [154]. Further analysis of cell lines and patient samples would elucidate such discrepancies.

### Urogenital tumors

TGF-β is a crucial molecule in the genesis of urogenital tumors, such as urinary bladder carcinoma, renal cell carcinoma, ovarian and prostate cancers [155].

The TGF-β pathway is involved in urinary bladder cancer progression. The amount of secreted TGF-β1 correlates with more aggressive phenotype of cell lines. In addition, deregulated TGF-β signaling led to enhanced migration and invasiveness of bladder cancer cells [156]. Silencing of TβRI expression by siRNA led to significant inhibition of TGF-β-induced signal transduction and thereby reduced invasiveness of bladder cancer cells [157].

Clear cell renal cell carcinoma (CCRCC) is the most common malignancy of the kidney; it accounts for 2-3% of all malignant diseases in adults [158]. In CCRCC patient samples, sequential loss of TβRIII and TβRII expression was associated with renal cell carcinogenesis and progression [155]. Cross-talk between Notch signaling and TGF-β pathway contributes to aggressiveness of CCRCC. Recently, it was described that inhibition of Notch signaling leads to attenuation of basal TGF-β-induced signaling in CCRCC cells; it also influenced genes involved in cancer migration [159].

### Ovarian cancer

In advanced ovarian tumors, low expression of TGF-β1 mRNA is connected to better prognosis. It was found that TGF-β1 mRNA expression was significantly lower in tumors of patients who had optimal surgery than in patients with suboptimal surgery. TGF-β1 mRNA expression was also significantly lower in tumors with high sensitivity to chemotherapeutics than in those with low sensitivity [160].

Alterations in the *TβRI* gene occur in ovarian cancer and account, at least in part, for the frequent loss of TGF-β responsiveness of these cancer cells. Presence of TβRI 6*A allele in about 27% of human ovarian cancers suggests that it acts as a low penetrating tumor marker in the development of ovarian cancer [161-163].

Mutations in the *TβRII* allele that cause loss or decrease in TβRII protein level are also present, BAT-RII mutations (mutations in polyadenine tract in exon 3) were found in 22% of ovarian tumors [161]. Although this mutation is connected to microsatellite stability, in ovarian cancers this association remains controversial [164].

Mutations in SMAD4 are not very common in ovarian cancer but were reported in primary cultures or cell lines [165]. Reduced expression or loss of SMAD4 protein leads to decreased ability to bind DNA; SMAD4 inactivation is involved in the acquisition of a more aggressive tumor [161].

It has been suggested that SMAD4 and SMAD3 are involved in metastatic potential of ovarian cancers [166,167]. In ovarian cancer cell lines, TGF-β supported metastatic activity at least partly through activation of MMPs [168]. Deregulation in TGF-β/SMAD4 signaling leads to epigenetic silencing of a putative tumor suppressor, RunX1T1, during ovarian carcinogenesis [169]. Recently, genome-wide screening done by ChIP-seq of TGF-β-induced SMAD4 binding in epithelial ovarian cancer revealed that SMAD4-dependent regulatory network was strikingly different in ovarian cancer compared to normal cells and was predictive of patients survival [170].

### Prostate cancer

In prostate cancer, high level of TGF-β1 expression is linked to tumor progression, cell migration and angiogenesis [171]. In some prostate cell lines, even low level of TGF-β1 induced its own expression in an autocrine manner. However, only in benign cells, higher concentration of TGF-β1 leads to recruitment of protein phosphatase 2A (PP2A) by activated TβRI, which terminates the induction of TGF-β1. On the contrary, in malignant cells, incorrect recruitment of PP2A by TβRI is responsible for protruded production of TGF-β1 [172].

When compared to other types of cancer, such as breast and colon, down-regulation of TβRs is found more often than mutations in SMADs. Kim *et al.* compared protein levels of TβRI and TβRII in benign and malignant prostate tissues and observed that loss of receptors expression correlated with more advanced tumor [173]. Decreased level of receptor protein is accompanied with decreased mRNA expression; thereby, loss of receptor expression is a potential mechanism to escape the growth-inhibitory effect of TGF-β [174]. However, mutations are present in only some cases of prostate cancer, which suggests that other mechanisms are involved. For example, in a study by Turley *et al.,* loss of TβRIII expression correlated with disease progression [175]. In some cases of prostate cancer, insensitivity to TGF-β is caused by promoter methylation in TβRI [176].

So far, mutations in SMAD2 proteins were not found in prostate cancer. However, studies *in vitro* revealed that SMAD2 functions as a tumor suppressor of prostate epithelial cells. It is possible that tumor suppressor function of SMAD2 could be lost during differentiation of normal tissues or during prostatic carcinogenesis [177-179].

### Breast cancer

In normal mammalian breast development, all TGF-βs isoforms are functionally equivalent; they are all involved in establishing proper gland structures and apoptosis induction. However, they have distinct roles in mammary growth regulation, morphogenesis and functional differentiation [180-182].

In breast cancer, results evaluating TGF-β as a prognostic factor are controversial. On the one hand, analysis demonstrated TGF-β1 expression to be significantly higher in patients with a favorable outcome as compared to patients with a poor prognosis [183]. On the other hand, several studies showed that TGF-β over-expression is related to worse outcome [184,185]. Elevation of TGF-β has been shown to participate in breast cancer metastasis [186].

Alterations of TGF-β signaling molecules are relatively rare, except for TβRII down-regulation. No specific mutations were found in the coding or in the regulatory region of the TβRII gene promoter in breast cancer [187,188]. However, the loss of TβRII expression has been linked to tumor progression and metastasis, principally in HER2-negative patients [114]. In addition, resistance of breast cell lines to TGF-β may be due to reduced expression of TβRII [189]. Mutations of TβRII are rare among breast cancer patients, while changes in receptor expression may take part in tumor progression [187]. Opposite to TβRII, intragenic mutations occur in TβRI and are associated with metastatic breast cancer [190].

Although the role of TβRIII remains unclear, it seems that this receptor is a suppressor of breast cancer. Loss of TβRIII through allelic imbalance is a frequent genetic event during human breast cancer development that increases metastatic potential; moreover, decreased TβRIII expression correlates with decreased recurrence-free survival in breast cancer patients [191].

Mutations in downstream signaling pathway including SMAD proteins are not very common in breast cancer; however, inactivating mutations or loss of expression in SMAD4 have been described [164,192].

### Tumors of the digestive tract

#### Gastric cancer

**Figure 5:**
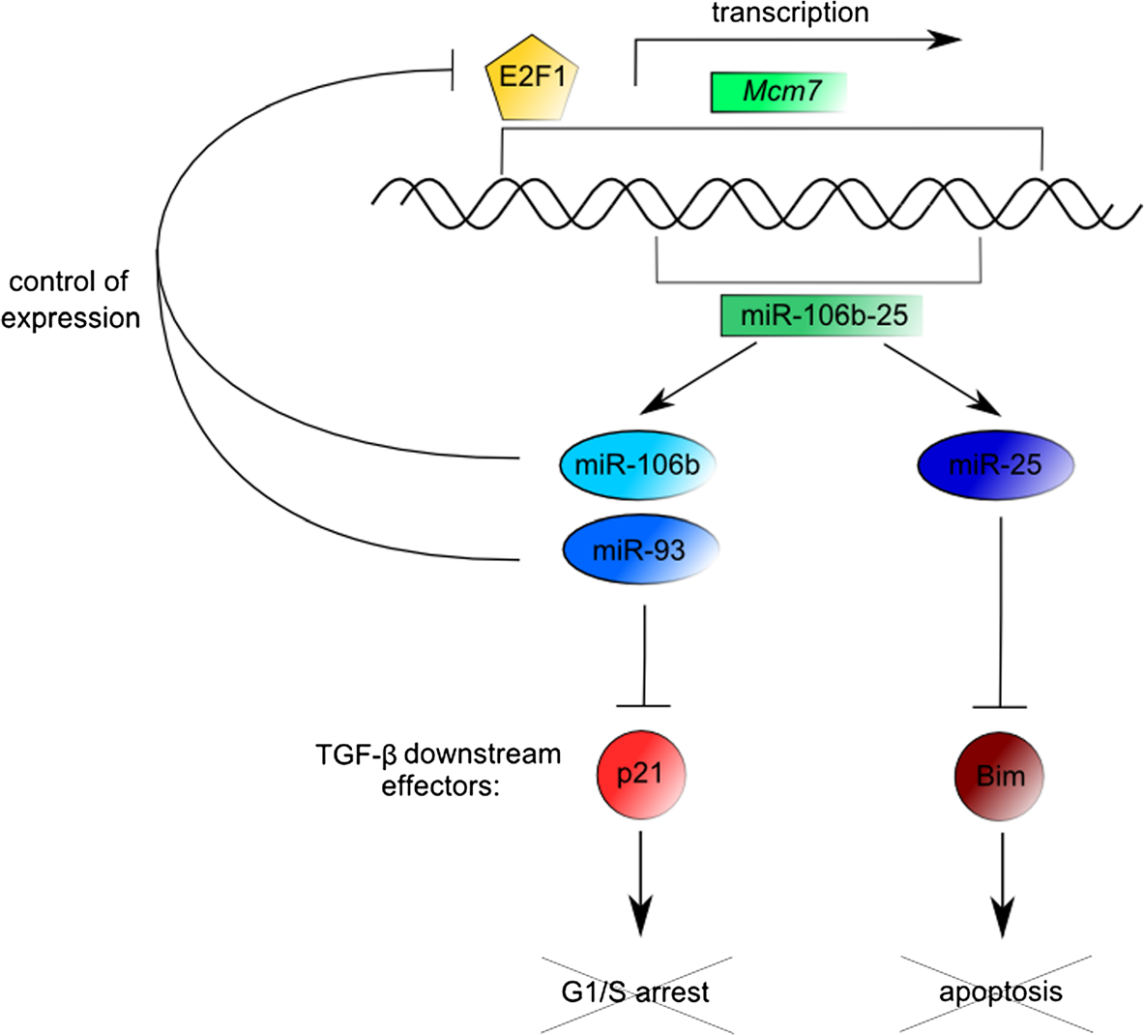
**The E2F1/miR-106b-25/p21 pathway.** In gastric cancer, miR-106b-25 cluster is activated by E2F1 in parallel with its host gene, Mcm7. In turn, miR-106b and miR-93 regulate E2F1 expression, establishing a miRNA negative feedback loop. Over-expressed miR-106b, miR-93, and miR-25 inhibit the synthesis of p21^CIP1^ and Bim (TGF-β downstream effectors) and therefore prevent cell cycle inhibition and apoptosis

Resistance to TGF-β is a hallmark of gastric cancer. The relationship between TGF-β resistance and up-regulated level of miR-106b-25 cluster (miR-106b, miR-93, and miR-25) has been recently elucidated [193]. The cluster is an intronic part of the *Mcm7* gene and thus is regulated by E2F1. Conversely, miR-106b and miR-93 control E2F1 expression thus establishing negative feedback that prevents E2F1 self-activation. Over-expression of miR-106b, miR-93 and miR-25 decreases response of gastric cancer cells to TGF-β since they interfere with synthesis of TGF-β downstream effectors that promote cell cycle arrest and apoptosis, such as p21^CIP1^ and BIM, respectively [193] (Figure 5). 

Mutations in TβRII that lead to insensivity of cell lines to TGF-β mediated growth inhibition have been previously described [194]. It has been shown that conditional loss of TGF-β signaling due to dominant negative mutation in TβRII leads to increased susceptibility to gastrointestinal carcinogenesis in mice [195].

Epigenetic changes in TβRI are another important mechanism of escape from TGF-β physiological function. Hypermethylation of a CpG island in the 5' region of the TβRI was found in 80% of gastric cancer cell lines and 12.5% of primary tumors. Treatment with demethylating agent increased expression of TβRI and transient transfection of TβRI into TGF-β resistant cell line restored TGF-β responsiveness [123].

Effects of TGF-β on gastric cancer invasiveness and metastasis are mediated by activation of JNK and ERK pathways which support expression of fascin-1, an actin-binding protein. Moreover, signaling pathway based on SMAD proteins is not involved in this process because transitional repression of SMADs did not alter fascin-1 expression [196].

Nevertheless, impaired signaling based on SMAD proteins also occurs in gastric cancer. Shinto *et al.* found a correlation between expression level of p-SMAD2 and patients prognosis. P-SMAD2 protein expression level was significantly higher in patients with diffuse form of carcinoma and metastatic tumors and is associated with worse outcome [197]. TGF-β signaling is also abrogated by decreased expression of SMAD3. Low or undetectable level of SMAD3 was observed in 37.5% of human gastric cancer tissues. In cell lines, which showed deficient expression of SMAD3, introduction of *SMAD3* gene led to growth inhibition caused by TGF-β [198].

Sonic hedgehog (Shh), a member of the hedgehog signaling pathway, promotes invasiveness of gastric cancer through TGF-β-mediated activation of the ALK5–SMAD3 pathway. Higher concentrations of N-Shh (human recombinant form of Shh) enhanced cell motility and invasiveness in gastric cancer cells; moreover, treatment of cells with N-Shh led to enhanced TGF-β1 secretion, TGF-β-mediated transcriptional response, expression of ALK5 protein and phosphorylation of SMAD3. Effect of Shh on cell motility was not observed after treatment of cells with anti-TGF-β blocking antibody or TGF-β1 siRNA [199].

#### Hepatocellular carcinoma

Reduced TβRII expression was observed in approximately 25% of hepatocellular carcinoma (HCC) patients; this event is associated with aggressive phenotype of HCC and intrahepatic metastasis. TβRII down-regulation also correlated with an early recurrence time and higher grade of tumor suggesting that TβRII down-regulation is a late event in HCC development. In addition, TGF-β is a tumor suppressor in the majority of HCCs expressing TβRII [200].

Mutations in intracellular signaling components have been observed: SMAD2 mutations occur in 5% of HCC, while loss of SMAD4 expression was found in 10% of HCC [201,202].

Several studies of HCC indicated that over-expression of SMAD3 promotes TGF-β-induced apoptosis [203,204]. Pro-apoptotic activity of SMAD3 requires both input from TGF-β signaling and activation of p38 MAPK, which occurs selectively in liver tumor cells. SMAD3 represses transcription of an important apoptotic inhibitor, BCL-2, by directly binding to its promoter [203].

Therapeutic options for patients with HCC are still limited; however, it was recently described that blocking the TGF-β signaling pathway with LY2109761, a kinase inhibitor of TβRI, is associated with inhibition of molecular pathways involved in neo-angiogenesis and tumor growth. LY2109761 interrupts the cross-talk between cancer cells and cancer-associated fibroblasts, leading to significant reduction of HCC growth and dissemination. Currently, LY2109761 is being tested in clinical trial phase II [205-207].

#### Colorectal cancer

In colorectal cancer (CRC), TGF-β1 inhibits proliferation of less aggressive tumor cells but stimulates growth of tumor cells at later stages by autocrine manner. High level of TGF-β1 correlates with tumor progression [208]. In colorectal cell lines, TGF-β induces proliferation by RAS-independent manner [209]. In a recent study, TGF-β, TβRI, TβRII, SMAD4, pSMAD2/3 and E-cadherin were found to be closely related to TNM stage of CRC. Therefore, TGF-β, TβRII, SMAD4, pSMAD2/3 and E-cadherin come into view as valuable independent biomarkers of prognosis in CRC patients [140].

Inactivating mutations in SMAD2 and SMAD4 are frequent especially in pancreatic and colorectal carcinomas, although they do not stand for the most frequent tumor changes. Most of SMAD2 mutations have been found in the MH2 protein domain, thereby preventing complex formation with SMAD3 and SMAD4. Alterations of SMAD2 are present in about 6% of colorectal carcinoma cases [210]. SMAD3 mutation is a very rare event in human solid tumors; however, a missense mutation in *SMAD3* gene (leading to reduced activity of SMAD3 protein) was found in human colorectal cell lines [211]. Inactivation of SMAD4 is a genetically late event in gastrointestinal carcinogenesis. It was identified with less frequency in advanced colon cancers and in 16% of colon carcinomas [212,213]. Nevertheless, recent studies revealed that some of the TGF-β induced pathways are SMAD4 independent [214]. Proteomic screen of *SMAD4* wt and *SMAD4* deficient cell lines detected different protein levels in cell lines pointing to SMAD4 dependent and independent TGF-β responses in colon carcinoma cells [215]. Another study indicated that novel genetic variant -4 T(10) in the *SMAD4* gene promoter affects its activity. Obtained preliminary results indicate that *SMAD4* gene promoter haplotype -462 T(14)/-4 T(10) represents a potentially relevant genetic marker for pancreatic and colorectal cancer [216]. This downstream inactivation of TGF-β signaling components promotes colon adenoma to carcinoma progression.

Mutations of TβRII are frequent alterations of the TGF-β signaling pathway (reviewed in [217]). They are present in approximately 30% of CRC cases and were reported in cancer cell lines, sporadic colon cancers and patients with hereditary non-polyposis colorectal cancer with microsatellite instability and in a smaller percentage in microsatellite stable cancers [123,218,219]. TβRII mutations occur in >90% of microsatellite unstable (MSI) colon cancers and most principally affect a polyadenine tract in exon 3 of TβRII, the BAT-RII; however, non-BAT point mutations in TβRII were found with less frequency also in microsatellite stable cancers [164,219]. Interestingly, it has been recently published that restoration of TβRII in cancer cell lines with microsatellite instability (MSI), bearing mutated TβRII, promoted cell survival and motility. Therefore, it is plausible that such mutations contribute to favorable outcome in MSI patients [220].

In contrast to TβRII, mutations in TβRI are less common. They are rare in colon as well as pancreatic cancer. Decreased TβRI allele expression is associated with higher risk of colon cancer development [221]. Recently, it has been described that TβRIII mRNA expression is not significantly altered in human colorectal cell lines; however, protein levels of TβRIII are frequently increased, suggesting a distinct role for TβRIII in colon cancer. Thus, enhanced expression of TβRIII is possibly involved in cancer progression [222].

Other mechanisms, such as crosstalk between TGF-β and Wnt/β-catenin pathways, are involved in colon cancer progression [214]. It has been shown that SMAD4 restoration is associated with suppression of Wnt/β-catenin signaling activity, decrease of β-catenin/Tcf target genes expression and with induction of functional E-cadherin expression [223].

Recently, the role of microRNA in colon cancer has been established. Elevated levels of miR-21 and miR-31 promote motility and invasiveness of colon cancer cell line and enhance the effect of TGF-β. It seems that miR-21 and miR-31 act as downstream effectors of TGF-β [224].

#### Pancreatic cancer

Pancreatic cancer has the poorest prognosis among GI cancers due to aggressiveness, frequent metastases and resistance to treatment. SMAD4, also called DPC4 (deleted in pancreatic carcinomas), suggests close relationship between loss of this gene and pancreatic cancer. Mutation or deletion of SMAD4 is a well-characterized disruption in the TGF-β pathway – it occurs late in neoplastic progression, at the stage of histologically recognizable carcinoma. In pancreatic cancers, SMAD4 is homozygously deleted in approximately 30% of cases, inactivated in 20%, while allelic loss of the whole 18q region was found in almost 90% of cases [225]. These mutations are present mostly in the MH2 domain; however, missense, nonsense or frameshift mutations are present within the MH1 domain as well [226,227].

Dual role of SMAD4 was established in a mouse model. *Smad4* or *TβRII* deletion in pancreatic epithelium did not affect pancreatic development or physiology. However, when activated K-Ras was present in cells, loss of *Smad4* or *TβRII* or *Smad4* haploinsufficiency led to progression to high-grade tumors. Thus, it is possible that Smad4 mediates the tumor inhibitory action of TGF-β signaling, mainly in the progressive stage of tumorigenesis [115].

In concordance with colorectal cancer, mutations in TβRII were found in cancers with microsatellite instability; however, mutations in TβRII and also in TβRI are less common [217]. Frequency of mutations in *TβRII* is about 4% and even less for *TβRI*[228]. Interestingly, polymorphism within the *TβRI* gene, which is less effective in mediating anti-proliferative signals than wild type, was described [229].

High level of TGF-β was found in serum of patients with pancreatic adenocarcinoma suggesting that TGF-β could possibly become a marker for monitoring disease activity [230].

As previously mentioned in HCC, targeting TβRI/II kinase activity in pancreatic cancer with the novel inhibitor LY2109761 also suppressed pancreatic cancer metastatic processes. LY2109761 suppressed both basal and TGF-β1–induced cell migration and invasion and induced anoikis. *In vivo*, LY2109761, in combination with gemcitabine, significantly reduced the tumor burden, prolonged survival and reduced spontaneous abdominal metastases [231].

#### Lung cancer

In non-small cell lung carcinoma (NSCLC), elevated expression of TGF-β correlates with disease progression [232]. Furthermore, significantly higher serum concentrations of TGF-β1 cytokine were found in lung cancer patients. Presumably, elevated expression and higher levels of serum TGF-β represent an important prognostic factor that could serve as a complementary diagnostic test in lung cancer detection [233].

Defective expression of TβRII was observed in primary NSCLC, where TβRII acts as a tumor suppressor. Down-regulation of TβRII on transcriptional level could be explained by aberrant methylation of the *TβRII* promoter [234]. Moreover, reduced expression of TβRIII has been found in NSCLC cells compared to normal human bronchial epithelial cells [235].

Downstream components of TGF-β signaling pathways are important in NSCLC development. Jeon *et al.* observed a correlation between better tumor-related survival and absence of SMAD6. Moreover, SMAD6 contributes to lung cancer progression by limiting TGF-β-mediated growth inhibition of cell lines, which was proven by knockdown of SMAD6 that resulted in increased apoptosis in lung cancer cell line [236].

TGF-β signaling is also required for lung adenocarcinoma (LAC) progression. In a study on LAC cell line A549, knockdown of TβRII resulted in suppression of cell proliferation, invasion and metastasis and induced cell apoptosis [237].

## TGF-β in hematological malignancies

### Leukemia

#### Myeloid leukemia

TGF-β is a potent inhibitor of human myeloid leukemia cells [238]. In acute myeloid leukemia (AML), t(8;21) translocation results in the formation of a chimeric transcription factor AML1/ETO. Jakubowiak *et al.* used transient transfection assays and a reporter gene construct that contained SMAD and AML1 consensus binding sequences and demonstrated that AML1/ETO represses basal promoter activity function and blocks response to TGF-β1. AML1/ETO possibly binds to SMAD3, instead of activating TGF-β1 signaling pathway. It represses TGF-β1-induced transcriptional activity and blocks TGF-β1 signaling, thus contributing to leukemia genesis [239].

In addition, in AML, dominant negative mutations in *SMAD4* were found. They are characterized by a missense mutation in the MH1 domain and a frameshift mutation in the MH2 domain of SMAD4. Mutated SMAD4 lacks transcriptional activity [240].

The t(3;21) translocation fusion product AML1/EVI-1 likely interacts with SMAD3 through the first zinc finger domain, represses SMAD3 activity by preventing SMAD3 from interacting with DNA, thereby repressing TGF-β-mediated growth suppression in hematopoietic cells. This way, AML1/EVI-1 contributes to leukemogenesis [241].

**Figure 6:**
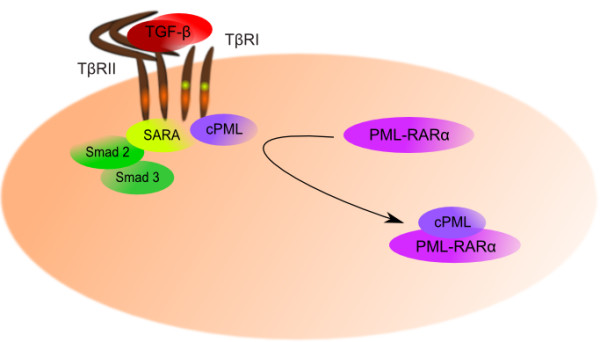
**TGF-β signaling in APL.** Cytoplasmic isoform of PML (cPML) protein interacts with SMAD2/3 and SARA and is required for accumulation of SARA-SMAD2/3 and TGF-β receptors in early endosome. However, the PML-RARα oncoprotein physically interacts with cPML and thus leads to impaired TGF-β signaling

In acute promyeloytic leukemia (APL), t(15;17) translocation in which the retinoic acid receptor α (RARα) gene on 17q12 fuses with a nuclear regulatory factor PML on 15q22 results in the fusion protein PML-RARα [242]. PML is normally found in 2 isoforms, a nuclear isoform and a cytoplasmic isoform. Cytoplasmic isoform is required for association of SMAD2/3 with SARA and for the accumulation of SARA and TGF-β receptors, resulting in SMAD phosphorylation (Figure 6). The PML-RARα oncoprotein antagonizes with cytoplasmic PML function by withdrawing cytoplasmic PML from the SMAD/SARA/TβRI/TβRII complex resulting in defects in TGF-β signaling [243]. 

In chronic myeloid leukemia (CML), t(9;22) (the so-called Philadelphia chromosome) results in the formation of BCR-ABL fusion gene [244]. The fusion protein is an active tyrosine kinase which enhances resistance of malignant cells to TGF-β-induced growth inhibition and apoptosis. BCR-ABL protein targets AKT and transcription factor FOXO3 and thus impairs the cytostatic effect of TGF-β1 [245]. In addition, by improving proteasomal degradation, BCR-ABL blocks TGF-β1-induced expression of p27^KIP1^. Thus, BCR-ABL kinase promotes activation of cyclin-dependent kinase and cell cycle progression [246].

In CML, expression of EVI-1, a proto-oncogene that is expressed at very low levels in normal hematopoietic cells, is increased. [247]. EVI-1 binds to the MH2 domain of SMAD3 repressing its DNA-binding ability and transcriptional activity and this way attenuates TGF-β signaling [248].

Moller *et al.* showed that BCR-ABL up-regulates TGF-β signaling when expressed in Cos-l cells. In Cos-1 cells, the expression of BCR-ABL up-regulates TGF-β-mediated transcriptional activity by interaction between TβRI and kinase domain of BCR-ABL, which leads to increased activity of SMAD3 promoter and increased SMAD2 and SMAD3 protein expression level [249].

#### Lymphoid leukemia

In children T-cell acute lymphoblastic leukemia (ALL), SMAD3 protein is absent or significantly decreased, however SMAD3 mRNA is present in T-cell ALL and normal T-cells at similar level. The level of SMAD3 is decisive for the T-cell response to TGF-β. A reduction in SMAD3 interplays with other oncogenic events, such as alterations in the retinoblastoma pathway, to precede T-cell leukemogenesis. It was proven that the loss of Smad3 can work in tandem with a loss of p27^KIP1^, which is also frequently altered in human T-cell ALL, to promote T-cell leukemogenesis in mice [250].

The t(12;21) translocation found in ALL generates the TEL-AML1 chimeric protein. Loss of sensitivity to TGF-β could be an important component of the function of TEL-AML1; it was shown that TEL-AML1 blocks the ability of TGF-β to suppress proliferation via activation of p27^KIP1^. The exact mechanism needs to be elucidated; however, a possible alternative is that TEL-AML1, in addition to binding SMAD3, binds co-repressors NcoR and SIN3A and this complex is able to transcriptionally activate the key cell cycle negative regulators, including p27^KIP1^[251].

Scott *et al.* showed that mRNA of downstream components of TGF-β pathway, such as p21^CIP1^ and p15^INK4B^, are absent in ALL cell lines with high frequency, while p27^KIP1^ mRNA levels are not reduced. These findings suggest epigenetic silencing of TGF-β signaling in molecular pathogenesis of ALL and possibly p15^INK4B^ and p21^CIP1^ are inactivated by this mechanism. In ALL, p15^INK4B^ mRNA absence is often connected with promoter methylation, whereas reduced p21^CIP1^ expression happens independently of promoter methylation, indicating that within the same malignancy, epigenetic silencing of TGF-β signaling is methylation-dependent or independent [252].

In adult acute T-cell leukemia, TGF-β signaling is inactivated through the activity of viral oncoprotein Tax. This oncoprotein compromises trans-activation of TGF-β responsive promoters by inhibiting the ability of SMAD proteins to mediate TGF-β-induced transcriptional activation by interfering with transcriptional factor CBP/p300 [253]. Another model of its function is that Tax interacts with the MH2 domains of SMADs 2, 3 and 4 in order to inhibit formation of the SMAD3/4 complex, disturb the interplay of the SMAD proteins with transcriptional factor CBP/p300, prevent binding of the SMAD complex to its target DNA sequence and thus inhibit TGF-β signaling [254]. The Tax repressor effect is mediated by activating JNK leading to increased phosphorylation of c-Jun, which is followed by formation of SMAD3/c-Jun complex that inhibits the ability of SMAD3 to bind DNA [255].

In hairy-cell leukemia (HCL), higher levels of TGF-β1 were observed in bone marrow (BM), serum and plasma from peripheral blood. The main source of this cytokine in active and latent form is hairy cell (HC). HCs produce TGF-β1, which is stored in BM near bone marrow fibroblasts; it activates them to synthesize collagen and reticulin fibers. TGF-β1 is important in fibrosis and is directly involved in the pathogenesis of BM reticulin fibrosis in HCL [256].

### Lymphoma

#### Peripheral and cutaneous T-cell lymphoma

In cutaneous T-cell lymphoma and Sézary syndrome, reduced levels of TβRI and TβRII correlate with decrease in TβRI and TβRII mRNA levels. This leads to the loss of TGF-β growth inhibitory responses [257].

Knaus *et al.* detected a single point mutation (Asp-404-Gly [D404G]) in the kinase domain of TβRII in advanced lymphoma. This dominant negative mutation prevents cell surface expression of normal TβRII. The ability of the mutant receptor to prevent function of normal TGF-β receptors is a new mechanism for loss of responsiveness to the TGF-β in tumorogenesis. Since TβRI is not able to bind TGF-β in the absence of TβRII, no TβRI is detected on the surface of these cells. This mutant receptor binds to normal receptor in an intracellular compartment, likely the endoplasmic reticulum, and blocks development of the normal receptor on the cell surface [258]. In addition, a 178-bp deletion in exon 1 in the gene for TβRI was reported to be responsible for loss of TβRI expression on the cell surface in anaplastic large cell lymphoma cell line JK. This deletion was confirmed to be present also in patients’ samples. Also, loss of TβRI is followed by loss of its tumor suppressive properties in human T-cell lymphoma [259].

#### Non-HodgkinÂÂ´s lymphomas (NHL)

ATL, adult T-cell leukemia/lymphoma is a rare form of Non-Hodgkin’s lymphoma (NHL). Zinc-finger E-box binding homeobox 1 (ZEB1) is a candidate tumor suppressor gene since mRNA of ZEB1 was found to be down-regulated in ATL. Physiologically, ZEB1 binds phosphorylated SMAD2/3 to enhance TGF-β signaling, and it can counteract the SMAD7-mediated inhibition of TGF-β1 function. Down-regulation of ZEB1 mRNA together with over-expression of inhibitory SMAD7 mRNA in ATL leads to loss of responsiveness to TGF-β-mediated growth arrest. Therefore, ZEB1 has an important role in regulation of TGF-β1 signaling pathway by binding to R-SMADs and also I-SMADs [260].

SMAD1 protein level is elevated and it is phosphorylated in response to TGF-β1 signaling in NHL. This suggests a role of SMAD1 in mediating the effects of TGF-β in NHL [261].

In B-cell lymphoma, Bakkebo *et al.* found that phosphorylation of SMAD1/5 is surprisingly an important event for the TGF-β-mediated anti-proliferative effects. TβRI was highly expressed in these cells and likely is important for signaling through SMAD1/5 pathway. Also, the regulation of TGF-β-mediated proliferation is at least partly dependent on activated p38 MAPK [262]. In B-cell lymphoma, the cell line resistant to TGF-β1 did not possess functional TβRII. This led to the absence of nuclear translocation of phosphorylated SMAD3 and SMAD2, the lack of nuclear expression of p21^CIP1^ and the down-regulation of c-Myc. Chen *et al.* found that methylation of promoter (CpG methylations at −25 and −140) plays an important role in TβRII gene silencing [263].

In diffuse large B-cell lymphoma (DLBCL), miR-155, which is over-expressed in aggressive type of B-cell lymphoma, targets SMAD5 by binding to the 3′ UTR of the SMAD5 gene. Treatment of DLBCL cell line with TGF-β1 resulted in phosphorylation of SMAD2/3 but also of SMAD1/5 indicating an active non-canonical signaling. Over-expression of miR-155 in this cell line significantly limited the cytostatic effect of cytokine due to impaired TGF-β1-mediated induction of p21^CIP1^. In miR-155-overexpressing and SMAD5 knockdown DLBCLs, the disruption of p21^CIP1^ induction was independent of the inhibitory effects of TGF-β1 thus creating a link between miR-155, TGF-β pathway and lymphomagenesis [264].

In small lymphocytic lymphoma/chronic lymphocytic leukemia (SLL/CLL), the CLL cells are resistant to the growth-inhibitory effects of TGF-β in spite of TβRII expression which is similar as in normal B cells. Therefore, the loss of responsiveness to TGF-β is most likely due to altered binding of TGF-β to the receptor complex or downstream signaling pathway [265].

Lagneaux *et al.* attributed the loss of responsiveness of CLL cells to TGF-β especially to decreased cell-surface expression of TβRI. CLL cells resistant to TGF-β1 showed no surface TβRI able to bind TGF-β1, but the expression of TβRII was normal. On the other hand, both TGF-β1-sensitive and TGF-β1-resistant CLL cells contained normal levels of TβRI and TβRII mRNAs. The absence of functional TβRI on the surface of CLL cells, in spite of normal mRNA level, could be explained by point mutations in the *TβRI* gene [266,267].

In CLL, Schiemann *et al.* found mutations in the signal sequence of TβRI (Leu12Gln substitution together with an in-frame single Ala deletion) which leads to reduced gene transcription stimulated by TGF-β [268]. In addition, CLL cells exhibited an increased expression of the TGF-β co-receptor, TβRIII, which is normally not expressed entirely in hematopoietic cells [269]. On the other hand, Lotz *et al.* found over-expression of TGF-β in CLL cells; all primary cells in this study were sensitive to the growth-inhibitory effects of this cytokine [270].

In BurkittÂÂ´s lymphoma, TGF-β-mediated growth arrest is associated with transcriptional repression of the *E2F-1* gene. On the other hand, over-expression of the *E2F-1* gene overcomes the TGF-β-mediated G1 arrest. So, the transcriptional repression of the *E2F-1* gene is required for growth arrest suggesting that TGF-β can effectively exert tumor suppression also in cells without c-Myc, p15^INK4B^ and p21^CIP1^ regulation [271]. Inman and Allday reported that in BurkittÂÂ´s lymphoma, cells express normal levels of TβRI RNA and protein, but decreased levels of TβRII RNA, leading to lack of responsiveness to TGF-β1 [272].

#### Multiple myeloma

**Figure 7:**
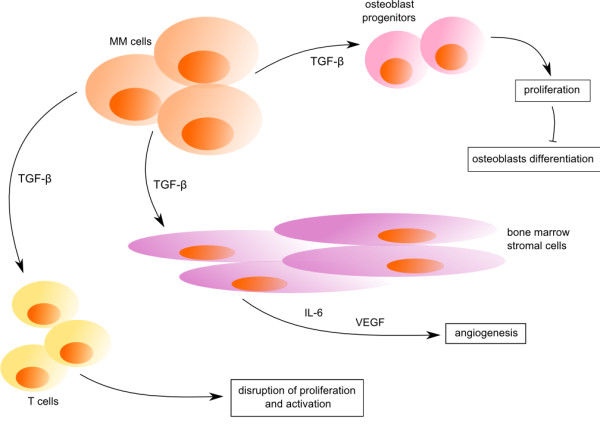
**TGF-β signaling in the bone marrow microenvironment of multiple myeloma.** Myeloma cells are able to produce TGF-β cytokine which influence cells of bone marrow microenvironment such as osteoblast progenitors, bone marrow stromal cells. Moreover, it disrupts T cell proliferation and activation

In multiple myeloma (MM), higher levels of TGF-β are secreted by myeloma cells as well as bone marrow stromal cells (BMSC). TGF-β secretion escalates with the stage of B cell differentiation (Figure 7). Increased production of TGF-β is followed by increased interleukin-6 (IL-6) and vascular endothelial growth factor (VEGF) secretion by BMSC, related to tumor cell proliferation. TGF-β is the major inducer of IL-6 and VEGF, two important cytokines of MM. On the other hand, TGF-β inhibits proliferation and Ig secretion of normal B cells [273]. 

After treatment with TβRI kinase inhibitor (SD-208), decreased production of IL-6 and VEGF and also attenuated tumor cell growth was observed. Mechanism of action of SD-208 is blocking nuclear accumulation of SMAD2/3 and related production of IL-6. This leads to inhibition of MM cell growth, survival, drug resistance and migration [274].

In MM, no mutations in *TβRI* or *TβRII* genes were described; MM cells contain TβRI and TβRII proteins in the cytoplasm. Resistance to the growth-inhibitory functions of TGF-β signaling develops, possibly due to defective trafficking of TβRI and TβRII to the cell surface in these cells [275,276]. Possibly, the loss of TβRII expression on the cell surface is the result of gene silencing by hypermehylation correlating to poor survival [277].TβRIII expression is diminished on mRNA and protein level in MM, enhancing cell growth, proliferation, mobility, heterotrophic cell–cell adhesion and contributing to disease progression [278].

Serum level of TGF-β is an important prognostic factor in MM. Higher levels of this cytokine mean lower levels of normal Ig resulting in immune impairment [279]. TGF-β secreted from MM cells disrupts proliferation, activation and IL-2 responsiveness in T cells. TGF-β is important in this immune-suppression, and its intensity of suppression is tumor burden dependent [280].

In MM patients, TGF-β represses bone formation in bone lesions. Initially, TGF-β enhances proliferation of osteoblast progenitors and promotes mineralization of bone matrix. Then, TGF-β inhibits subsequent phases of differentiation of osteoblasts and represses mineralization of matrix. This effect can be abrogated by inhibitors of TβRI kinase domain (reviewed in [281]).

## Conclusion

TGF-β signaling is complex and finely regulated fundamental pathway, which has an important role during human development and adult life. It is broadly intertwined with other signaling pathways. Moreover, it is involved in cancerogenesis of solid tumors as well as hematological malignancies. Paradoxically, TGF-β is both a tumor suppressor and tumor promoter. The tumor suppressor activities are widely described as anti-proliferative and apoptotic effects. During cancer progression, tumor frequently avoids tumor suppressive activities of TGF-β either by acquiring mutations of signaling components or by inhibiting its anti-proliferative response. This ’switch’ helps the tumor to use TGF-β as an oncogenic factor inducing tumor motility, invasion, metastasis and epithelial-to-mesenchymal transition. Advances in the study of molecular mechanisms that elucidate oncogenic activities of TGF-β lead to a strong desire to target TGF-β signaling in cancer therapy. However, the exact mechanisms involved in the malignant transformation of TGF-β needs to be clarified. Only then, it will be possible to develop successful therapeutic strategies as well as provide new therapeutic targets to restore the normal TGF-β function.

## Competing interests

The authors declare that they have no competing interests.

## Authors' contributions

L.K. wrote the original manuscript, L.S. and R.H. cooperated on revising the manuscript. S.S. revised the manuscript critically and approved the final version of the manuscript. All authors read and approved the final manuscript.
